# Determinants of screening participation of disadvantaged populations in France: a qualitative study

**DOI:** 10.12688/openreseurope.17317.3

**Published:** 2026-02-27

**Authors:** Alice Le Bonniec, Amandine Andrin, Alexandra Lelia Dima, Laurent Letrilliart

**Affiliations:** 1 Research on Healthcare Performance RESHAPE INSERM U1290, Universite Claude Bernard Lyon 1, Villeurbanne, Auvergne-Rhône-Alpes, France; 2 Groupe de Recherche en Psychologie Sociale (GRePS) EA4163, Universite Lumiere Lyon 2, Lyon, Auvergne-Rhône-Alpes, France; 3 National Screening Service, Dublin, Ireland; 4 Health Technology Assessment in Primary Care and Mental Health (PRISMA), Institut de Recerca Sant Joan de Déude Llobregat, Spain, Santa Rosa 39-57, Esplugues de Llobregat, 08950, Spain; 5 Centro de Investigación Biomédica en Red de Epidemiología y Salud Pública CIBERESP, Madrid, Spain; 6 Avedis Donabedian Research Institute (FAD), Barcelona, Spain; 7 Collège universitaire de médecine générale, Universite Claude Bernard Lyon 1, Villeurbanne, Auvergne-Rhône-Alpes, France

**Keywords:** disadvantaged population, deprivation, disability, health, screening, qualitative study

## Background

Screening is a secondary prevention practice that consists of early detection of diseases or risk factors for diseases with the aim of improving their prognosis. ^1^ In France as in many other developed countries, several screenings are recommended for the general population or for specific population subgroups. French screening programs are organized for particular cancers (i.e. colorectal cancer, breast cancer and cervical cancer) and free-access screening centers are dedicated to sexually transmitted infections and viral hepatitis. Depending on individual characteristics, health authorities recommend several screening procedures, such as for: cancers (colorectal cancer, breast cancer, cervical cancer); infectious diseases (HIV, Hepatitis C, syphilis, chlamydia infection); or cardio-metabolic problems (abdominal aortic aneurysm, diabetes, dyslipidemia, tobacco use, obesity).

However, strong social inequalities in health exist regarding access to recommended screenings, especially for the most disadvantaged people, as highlighted by a study conducted in France. ^2^ Figures on screening uptake within different French areas are scarce to illustrate screening disparities, but for example, data on cervical cancer screening shows that about 60% of under screened women lived in underserved areas. ^3^ Similarly, a French study exploring the social and territorial inequalities in breast and cervical cancers screening concluded that the more deprived the area of residence, the lower the screening uptakes. ^4^ Similarly, individuals with a low socio-economic level were less likely to be tested for cardiovascular disease risk according to a study conducted in the United Kingdom (UK), ^5^ for colorectal cancer according to a Spanish study ^6^ or for cervical cancer according to a Swedish study. ^7^ A study conducted in the UK also revealed socio-economic inequalities in screening for aortic aneurysms in men. ^8^ In addition, an American study found that people with disability were less likely to receive a recommendation for a cancer screening test. ^9^ This result is consistent with another study on screening for breast and colon cancer conducted in the UK. ^10^ In France, people living with a disability have also a low participation rate in cancer screening compared to the general population. ^11^

According to a framework developed to explore health screening in men, ^12^ barriers and facilitators to screening participation can be related to four domains (individual, social, healthcare system, screening procedure domains). In this framework, the individual domain includes the knowledge, perceptions and emotions of the individuals. The social domain reflects the influence of their social environment regarding screening. The healthcare system include factors related to the access to screening and the influence of healthcare professionals. The screening procedure factors concern the technical features of screening tests.

Previous studies conducted in France and internationally have explored barriers and facilitators of screening uptake ^13^^–^. ^15^ However, they have generally focused on screening for a particular condition (e.g., breast cancer screening). Thus, instead of a vertical disease-oriented approach common in evidence generation, it should be beneficial for preventive practice to adopt a more horizontal person-centred approach. ^16^ An umbrella review conducted with this approach and based on the framework previously mentioned ^12^ synthetized the existing literature on determinants to screening participation. Results showed that across conditions, screening participation was higher when the quality of patient-provider communication was better and when patients had adequate knowledge and positive beliefs about screening. Major barriers commonly cited were experiencing fear –of the discomfort of the testing procedure, of knowing the results and of having the disease-, feeling embarrassed, ashamed or stigmatized, and facing difficulties to access the procedure. ^17^ However, little is known about the particular knowledge, views and perceptions of people with a low-socio-economic background ^18^ or with disability ^19^ across screening conditions. The aims of this study were 1) to explore how people from deprived backgrounds or with disability experience the various recommended screenings, 2) to identify and classify the main barriers and facilitators to their participation in screening procedures by domain (individual, social, healthcare system, screening procedure domains), and to outline which domains have the most potential for intervention development.

## Methods

The COnsolidated criteria for REporting Qualitative research (COREQ) checklist was used to guide the reporting of the study. ^20^ The COREQ checklist is available as extended data I.

### Data collection

We conducted a qualitative study with semi-structured interviews. The interview guide included questions on health perception, screening perception, screening experience, factors motivating and hindering participation, and expectations towards screening procedures. Participants from deprived backgrounds and with disability were recruited via two public and four associative organizations providing social care services. People were eligible to participate if they were aged between 18 and 74 years; and if they were identified by the partner organizations as being from a deprived background (e.g. low socio-economic status, eligibility to receive subsidies from the government) or living with a physical or mental/intellectual disability. Exclusion criteria were as follows: people who were not competent French speakers and/or did not comprehend spoken or written French; people suffering from a serious disease (e.g. cancer being treated). Interviews were conducted face-to-face by two female researchers experienced in qualitative methods (ALB - PhD and AA – master’s student), in the partner organizations’ facilities or in another place at the convenience of the participants. The interviewer and the interviewee were alone. Interviewers had no prior relationships with participants. Participants were informed in written and verbally about the aim of the research, the data collection and analysis, and they were asked to sign an informed consent. Participants answered a brief socio-demographic questionnaire before the interview. A list of recommended screenings was presented to participants (available as extended data II) to help them identify which procedures they might have heard about or participated in. Interviews were recorded with participants’ consent. The level of social deprivation was assessed with the EPICES questionnaire, ^21^ with a score ranging from 0 (no social deprivation) to 100 (maximum social deprivation) and a deprivation threshold set at 30. Both groups had average scores around 41.

The interviews were conducted between May and July 2019. A framework analysis was then performed on the corpus of transcribed interviews. The study was approved by the French Institute of Medical Research and Health (INSERM) Ethics Committee (CD/EB 19-020) on the 19 ^th^ of February 2019. Data collection started on the 17 ^th^ of May 2019. Written informed consent was taken from all the participants. All methods were performed in accordance with the relevant guidelines and regulations in the Declaration of Helsinki.

### Data analysis

A content analysis using an analytical framework was performed on verbatim transcripts using NVIVO software (NVivo QSR International v.10). ^22^ A structure of inductively- and deductively-derived themes was employed. The analysis was independently conducted by two researchers (ALB, AA) before being compared to resolve any disagreement. It focused on barriers and facilitators to screening participation, which were categorized in four domains, based on the framework of Teo *et al.* in 2016 on barriers and facilitators to health screening in men ^12^: individual domain (e.g. attitudes, emotions), social domain (e.g. family and peer influence), health system/healthcare professional domain (e.g. accessibility, communication with healthcare professionals) and screening procedure domain (e.g. nature of screening procedure). This framework was chosen to guide the qualitative analysis considering that it covers all the screening domains of interest. Barriers and facilitators were identified and labelled within these categories inductively.

### Validity procedures

The design of the study was created in accordance with recommendations about validity of results. ^23^ More precisely, credibility was satisfied by recruiting participants through various organisations to achieve a diverse sample, and by the conduction of a systematic review about barriers and facilitators to participation in health screening across conditions. ^17^ Authenticity was satisfied by the achievement of interviews until the data collected was judged to be adequate in both amount and variety to answer the research question. Concerning transferability, the interview process has been detailed, and the interview topic guide (in French) is available upon request. Finally, concerning confirmability, two researchers individually coded the framework analysis.

## Results

### Sample

In total, 30 semi-structured interviews were conducted with 18 participants from deprived backgrounds and 12 participants with disability (20 women and 10 men, with an average age of 45 years). Interviews lasted between 15 minutes and 1 hour. Characteristics of participants are presented in Table 1. A majority of participants was unemployed. All participants had a referent general practitioner (GP). Most of them had already participated in at least one screening.

**Table 1.** Characteristics of the sample.

	Deprived population n = 18	Disabled population n = 12	Total n = 30
**Sex**			
Male	3	7	10
Female	15	5	20
**Average age**	43.1	48.0	45.3
**Family status**			
Married/In a relationship	10	3	13
Single	3	7	10
Divorced/Separated	5	2	7
Widowed	0	0	0
**Socio-professional category**			
Farmer	0	0	0
Artisan, trader or entrepreneur	0	0	0
Senior executive or intellectual profession	0	1	1
Intermediate occupation	0	0	0
Liberal profession	0	0	0
Employed	0	2	2
Worker	0	0	0
Retired	2	3	5
Unemployed	16	6	22
**Work or worked in the health field**	7	3	10
Have a referent GP *	18	12	30
**History of screening**	16	12	28
**Current chronic disease**	5	9	14
**Previous serious condition**	3	2	5
**Family history of serious condition**	9	10	19
Average EPICES score **	41.1	40.6	40.9

* GP: general practitioner.

** EPICES score: questionnaire to assess the level of social deprivation.

### Screening experience

Most participants from deprived backgrounds (n = 11) and 5 participants with disability mentioned that they had accessed to screening after having received a recommendation from a healthcare professional and/or from assistants or educators for people living in foster home.

On the contrary, 7 participants from deprived backgrounds and 4 participants with disability said they participated in screening procedures at their own initiative.

Six participants from deprived background stated that they had never received any screening recommendation from a healthcare professional.

### Main barriers and facilitators to screening participation

Main barriers and facilitators to screening participation are presented by domain (individual, social, healthcare system/healthcare professional, screening procedure) with illustrative quotes in Table 2, for both groups. A model illustrating the findings is presented in Figure 1.

**Table 2.** Barriers and facilitators to screening participation by domain and by population.

Factors Barriers 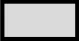 Facilitators 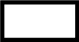	Population from deprived background (N=18)	Population with disability (N=12)	Examples of quotes
**INDIVIDUAL DOMAIN**			
No barrier	5	7	“ *I don’t see what could prevent me to get screened. I don’t think about something logical*” (man, with disability, 32 yrs)
Fear of screening	5	3	“ *Well, we are always afraid, I will not say the opposite [laughs]. Yes, we are afraid of certain things.”* (woman, deprived background, 42 yrs) *“That's because I'm afraid of the result. Even though I know there's a ninety-nine percent chance that I have nothing, there's always that little risk, so I prefer not to go.”*(man, with disability, 34 yrs)
Lack of time (e.g. caring responsibilities, other priorities)	3	-	“ *No I don't have time, it's important, but I don't have time, the doctor already gave me recommendation for the breast, I tried to find the time to go, but no I don't have time.*” (woman from deprived background)
Taking care of own health	7	3	“ *It is better to know what you have and also not to let it drag and become very serious.”* (woman, deprived background, 34 yrs) *“I get screened because I don’t want to suffer”* (man with disability, 51 yrs)
Wanting to know	5	1	“ *Yes it's always good [to get screened], we have nothing to lose on the contrary. So frankly it's always good for us, knowing what we already have*”. (woman from disadvantaged background, 37)
Fear of disease as a motivator	4	-	“ *What encourage me to get screened? Well, you could say that I'm someone who's afraid of catching something*” (woman from deprived background)
**SOCIAL DOMAIN**			
Social influences	9	3	“ *Honestly, I think that if I had not had a lot of people who had become ill around me, maybe I would not feel concerned by the screening question.”* (woman from deprived background, 34 yrs) *“Now that I have a little girl, I think I'm more aware about my health, because I know I'm not alone now, there's someone who depends on me. And I think it's really important for me to take care of my health. Because of her.”*(woman from deprived background, 31 yrs)
To meet a new partner (specific for HIV screening)	-	4	“ *Well, if we have sexual intercourse, I want to know if the person is sick. Because I don't want to have this [HIV].”* (man with disability, 36 yrs)
**HEALTHCARE SYSTEM/HEALTHCARE PROFESSIONAL**			
Not experiencing symptoms	4	-	*“I don’t think I'll do anything until I know, before I get hurt.”*(woman, deprived background, 36 yrs)
Do not feel concerned/at risk	4	4	“ *[Regarding sexual infections] It depends if we trust. I trust my husband, so for my part, there is no problem. The same for chlamydia I think.*” (woman from deprived background, 34 yrs)
Fatigue if too many medical procedures or addition of a supplementary constraint	3	2	“ *No, I'm not going to live like this, as I lived all my youth already, to undergo investigations, with pipes in the mouth, in the lungs. I did enough when I was young, when I will be old, I don’t want this anymore*”. (woman from deprived background, 74 yrs) « *My life is hard enough as it is so I don't want to stress myself out with other things.* » (man with disability, 34 yrs)
Feeling concerned (at risk)	7	3	“ *I talk more [about screening] to the doctor because it’s true that when we get older, well, it’s true, we want to know.*”(woman from deprived background, 42 yrs) “ *The prostate, I'm fifty-one years old, soon fifty-two, so you have to do all the assessments. And then a blood test too, because that's how you can detect it early on if you have cancer. Especially since in my family, there are four people who died from cancer. So we are subject to this.*” (man with disability, 51 yrs)
Recommendation from a healthcare professional	4	5	“ *It was my general practitioner who prescribed my blood test prescription. He had offered me a blood test for blood sugar and to check if I had diabetes or not. And everything was fine with the result.*” (woman with disability, 46 yrs)
Screening seen as inevitable when followed up for a disease/medical procedure	4	3	“ *Systematically, with each operation, they do it to you [blood testing]. Even if you are negative and you tell them, they make you do it again.”* (woman with disability 34 yrs) “ *There are some [screenings] that I did because it was an obligation, during pregnancies, it was compulsory, everything that is HIV is forced, and other diseases that we are forced to do also.*"(woman from disadvantaged background, 42 yrs)
Trust in healthcare professionals	-	4	“ *I trust the medical profession. So if there is a screening to do, I do it.*" (man with disability, 51 yrs)
**SCREENING PROCEDURES DOMAIN**			
Screening procedure difficult to access/perform	-	3	“ *I haven’t done it [screening for cervical cancer] these days because I have a very narrow cervix and you can’t remove the smear, it’s very complicated.*” (woman with disability, 46 yrs) “ *I’m in an electric chair, and if the elevator doesn’t work … I’m staying at home, so I can’t go to the laboratory. But what I can do, it happened to me, I cancel the appointment. I ask to the doctor to make a prescription to bring the laboratory to my house. And there you go. That happened to me once.*” (man with disability, 51 yrs)

**Figure 1.**
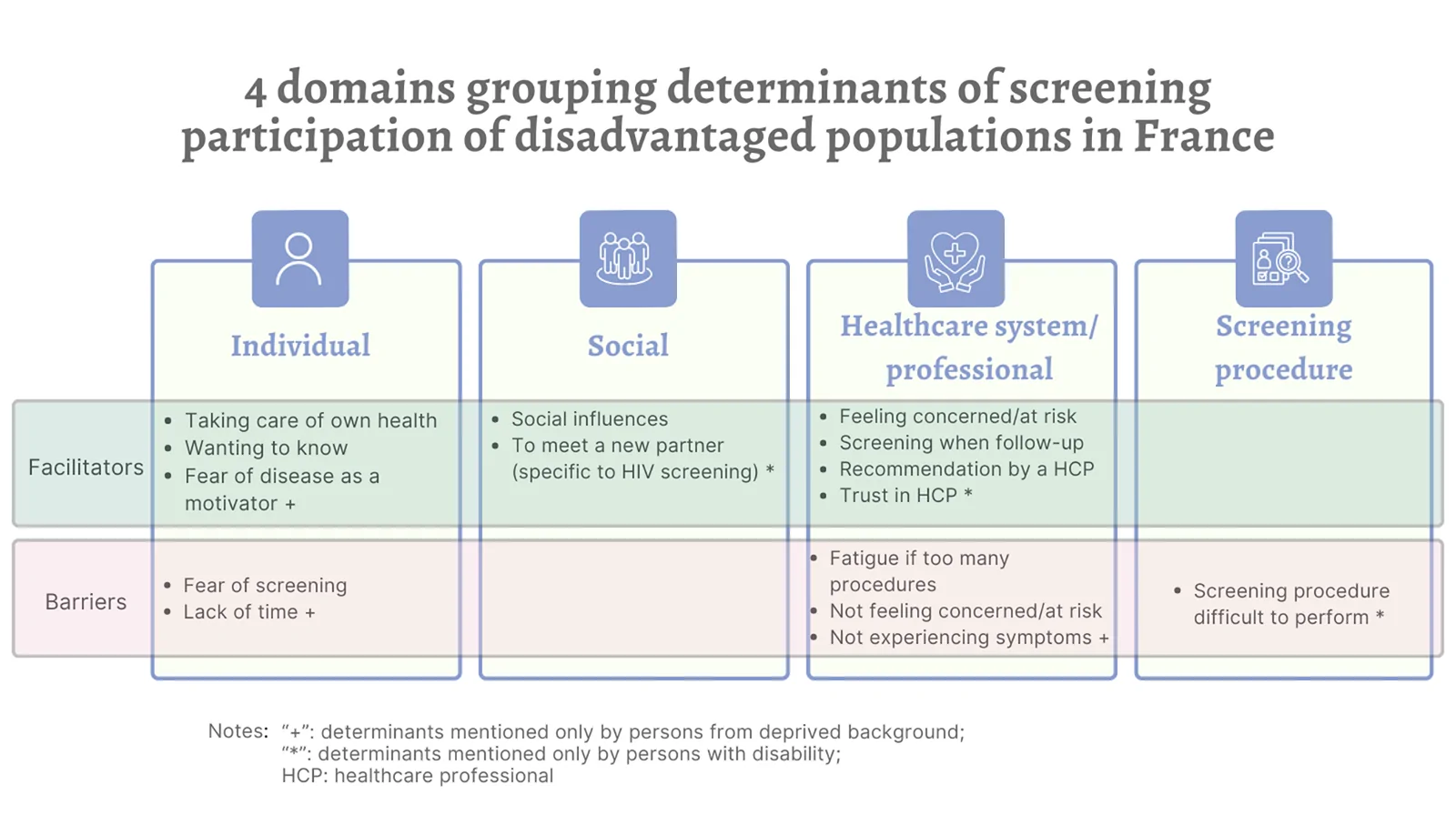
Model illustrating the findings.

Participants from deprived backgrounds cited more barriers to screening participation (n=6) than participants with disability (n=4). Conversely, people with disability cited more facilitators (n=8) than those from deprived background (n=7). Most barriers and facilitators to screening participation were related to the individual domain and to the healthcare system/healthcare professional (or educator) domain.

In the individual domain, *fear* was reported to have a negative influence on screening participation for both groups, but *fear* could be also a *motivator* for people from deprived backgrounds. Another barrier mentioned only by participants from deprived backgrounds was *lack of time.* Some participants from both groups outlined that they faced *no barrier* to screening participation. *Taking care of one’s own health* and *wanting to know* were two facilitators cited by both groups.

The social domain included only facilitators. Half of the deprived background group and a few participants with disability mentioned *social influences* as a facilitator, through awareness from others’ experiences, or through the willingness to stay in good health to be able to take care of relatives. *To meet a new partner (specific for HIV screening)* was reported as a facilitator only for participants with disability.

In the healthcare domain, participants from deprived backgrounds reported *not experiencing symptoms*, *not feeling concerned or at risk*, and *fatigue if too many medical procedures or addition of a supplementary constraint* as barriers. Participants with disability also reported two of these barriers: *fatigue if too many medical procedures or addition of a supplementary constraint* and *not feeling concerned or at risk.* At the opposite, *feeling concerned or at risk*, a *recommendation from a healthcare professional (or educator)*, and *screening seen as inevitable when being followed up for a disease or medical procedure* were reported as facilitators by both groups. Two participants with disability highlighted the fact that they did not really have the choice to participate or not in screening (blood tests) because it was part of a care protocol related to their disability. *Trust in healthcare professionals* was only mentioned by participants with disability as a facilitator.

Finally, the screening procedure domain was only mentioned as a barrier for participants with disability as they could face *practical difficulties to access or perform screening procedures* (e.g. being in a wheelchair).

## Discussion

### Summary of results and comparison with other studies

This qualitative study collected the experiences, barriers and facilitators of all recommended screenings for people from deprived backgrounds and people with disability. Most barriers and facilitators to screening participation were related to the individual domain and to the healthcare system/healthcare professional (or educator) domain. The social domain included only facilitators (e.g. social influences). Participants with disability seemed to be more influenced by factors related to healthcare system/healthcare professionals. Finally, the screening procedure domain was only mentioned as a barrier for participants with disability.

***Population from deprived background.*** In the individual domain, fear was often mentioned by participants from deprived backgrounds. A distinction can be made between a fear of the results of the test (i.e. diagnosis, consequences of a disease) and a fear of the screening procedure (i.e. painful test). ^17^ In accordance with our findings, the review of Young *et al.* in 2018 ^24^ about cancer screening attendance in the UK showed that fear was described as being both a motivator and a barrier to screening participation. Worries about the consequences of an asymptomatic infection was also mentioned as a facilitator to chlamydia testing in a systematic review. ^25^ In our qualitative study, wanting to know one’s own health status was described as a facilitator by several participants from deprived background. This facilitator was also found in systematic reviews about HIV screening ^26^ and about cervical cancer screening. ^27, 28^

Participants from deprived backgrounds seemed to be strongly influenced by their social interactions, especially through experiences from others or social responsibility to stay in good shape to take care of family members. A mixed-method study performed in Bulgaria and Romania about barriers and women attitudes toward cervical cancer screening highlighted the importance of women’s perceptions of what other women in their social networks were doing and their attitudes toward screening. ^29^ The influences arising from family and close friends were also described as being of major importance in relation to women’s participation in mammography screening in a Greek qualitative study. ^30^

Interactions with healthcare professionals seem to be an important component of screening decisions. A Canadian qualitative study similarly mentioned the important role of trust in physicians in homeless women’s decision-making about breast and cervical cancer screening. ^31^ Consistent with our findings, receiving a physician’s recommendation for screening was reported in a systematic review as a facilitator for both breast and cervical cancer screening for indigenous populations. ^32^

***Population with disability.*** Our results revealed that more than half of the participants with disability did not report experiencing any barrier to screening participation. In the individual domain, fear was the only barrier mentioned. In line with our results, feelings of anxiety and fear about cancer screening programmes were expressed by women with a learning disability according to a systematic review about cancer screening in UK. ^33^

According to our results, social interactions have a positive influence on screening participation for people with disability, and particularly regarding HIV screening. For example, some participants with disability expressed a willingness to have a HIV test in the context of a new (potential) partner. Similarly, testing to protect a partner or because a partner was HIV-positive or at high-risk was a facilitator also reported in a systematic review about barriers and facilitators to HIV testing. ^26^

Findings suggest that participants with disability have more links with the medical community and healthcare professionals, and this may have a positive impact on their screening practices. Nevertheless, the notion of free and informed consent can be questioned in such a context. Indeed, two participants expressed a perception of imposed screening (blood tests) as part of another medical procedure. This specific context places screening as an inevitable step to access other care, whereas informed consent should be checked for each separate procedure. Likewise, it is possible that people with disability, often confronted with medical procedures, are more inclined to agree to participate in screening (or quite simply not to ask questions) because they are used to having medical procedures.

According to our results, difficulties in accessing or performing the screening procedure was the only barrier in the screening procedure domain. Similar findings were found in systematic reviews ^34, 35^ about barriers and facilitators to cancer screening for adults with a physical disability, in which a lack of access to screening was outlined.

In our study, already having too many medical investigations or addition of a new constraint were mentioned as barriers by people with disability but also by people from deprived backgrounds. Interestingly, a review examining cancer screening participation for adults with a physical disability identified a barrier which was unique to disabled women: their perspective that having to address their pre-existing conditions was enough to deal with. ^35^ This common results for different populations might reflect a thin separation between our two groups: people from disadvantaged backgrounds can also face important health issues, and participants with disability can also have a low socio-economic level.

### Strengths and limitations

This study helps to understand the perspectives on screening of two understudied populations - people from deprived background and people with disability. In addition, by studying various recommended screening procedures at the same time, this research considers a horizontal approach to participation in screening, which differs from the vertical approaches usually addressed in the literature (i.e. focusing on a single screening condition). This qualitative study presents also several limitations that should be highlighted. First, the number of participants was limited, due to the difficulties to have access to disadvantaged populations. However, interviews were conducted until reaching data saturation in both groups. Second, all interviews were conducted in the same metropolitan area. It is possible that variations in findings could be found with participants from other areas. Similarly, only French speakers were included and data on ethnicity was not collected. Third, participants were recruited from charities and non-profit organizations, implying that they were in contact with those structures. Consequently, we did not have access to people living in more isolated contexts, who might face different barriers to accessing care and participating in screening. In addition, 28 out of 30 study participants had already participated in at least one screening. However, it is likely that only a few or none of them had participated to all screening recommended to them. Fourth, the level of health literacy was not assessed during the interviews. Finally, there was no public and patients involvement in the study design nor in the interpretation of the study outcomes.

### Implications and perspectives

An integrated horizontal person-centered screening participation approach could be applied to screening recommendations for disadvantaged populations. According to clinical practice guidelines, healthcare professionals should recommend all screening procedures relevant to their patients, according to their profile ^36, 37^ . Previous work showed the value of adopting a common approach across screening conditions. ^17, 38, 39^ Identifying the recurrent barriers to screening across conditions can serve as a basis for intervention development to support shared decision-making, and has also the potential to be used for training primary care professionals. ^17^ At a societal level, horizontal screening campaigns have already been developed in France such as the one encouraging people over 16 year who are far from the health system and in a precarious situation to attend a free and personalized preventive health exam. Including occupational medicine for short-term contracted workers or public services (e.g. unemployment, social welfare) in these campaigns could be an effective way of reaching disadvantaged populations.

On the basis of our findings, we can suggest several ways of improving screening participation for populations with disability and from deprived background. Our results can indeed be mapped to the Behaviour Change Wheel and associated Behaviour Change Techniques. ^40, 41^ Those techniques could be used to promote several screening procedures that are recommended for a same patient. For example, a woman aged 60 can be invited simultaneously for cervical cancer screening, breast cancer screening, osteoporosis testing and infectious disease screening. First, developing an intervention based on social opportunity seems to be relevant to build on the potential influence of relatives and friends that could help to improve screening participation across conditions. For example, using social support (e.g. encouragements from people who have already participated in screening), or using modelling (a role model) when developing awareness campaigns could be effective strategies. ^42^ Adopting a community-based approach based on a comprehensive approach including education and navigation seems also relevant to improve screening uptake. ^43^ Second, increasing psychological capability by shaping knowledge could be also relevant for intervention development and could be applied to all recommended screening. For example, outlining the benefits of screening for health preservation in clinical practice and awareness campaigns could be beneficial, as participants outlined willingness to take care of their own health. Third, interventions should focus on social environment and healthcare providers to increase motivation to participate in screening across conditions, since the main facilitators found in this study were related to those domains. Decision aids or shared decision making tools could rely on education and social support to encourage people to participate in all recommended screenings, according to their health and psycho-social profile. Indeed, having a long-term relationship with a primary care clinician and sharing decisions were identified as key factors to increase colorectal cancer screening uptake in a randomized controlled trial designed to test engaging underserved populations in preventive care through online material. ^44^ In Denmark, a decision aid tool successfully increased colorectal cancer screening uptake and positive screening attitudes among a lower educated population. ^45^ Fourth, increasing the physical capability using environmental restructuring (changing the context) is a key implication from our results. Accessibility to screening procedures should be improved for disadvantaged populations, especially for disabled people. For example, general health checks in adult primary care could help in this regard. ^46^

Finally, further research should explore more deeply the effects of fear on screening practices through various perspectives. For example, the regulatory focus theory proposed by Higgins ^47^ could be tested by developing different messages about screening, highlighting either gain or loss associated with screening participation (i.e. to avoid a disease vs. to promote health). In addition, future research should investigate the interactions between factors related to the individual, social and healthcare professional/educator domains. For example, both family, friends and healthcare professionals, as trusting persons, can provide information and reassurance to people confronted to fear of screening, and so can have a positive influence on screening participation.

## Conclusion

This qualitative study explored the views and perspectives on screening programs of two understudied populations: people from deprived background and people with disability. Findings highlighted the importance of the social environment and healthcare professionals to promote screening participation. Further research should focus on developing relevant communication and decision making tools to improve screening awareness, motivation and accessibility for disadvantaged populations.

## Data Availability

The datasets used and analysed during the current study cannot be shared openly due to data protection requirements in effect in France at the time of data collection. This research was conducted in accordance with GDPR regulations. According to the CNIL (French data protection authority), the authors are not allowed to reuse the data or transfer the data to someone outside the research team if participants had not been informed. When invited to take part in the study, participants only gave their consent for their data to be used for the purpose of the analysis performed by the study team and did not give their consent for further use. The data are archived electronically and access may be granted upon reasonable request to the corresponding author. Zenodo: Appendices - Determinants of screening participation of disadvantaged populations in France: a qualitative study – OpenResearchEurope, https://doi.org/10.5281/zenodo.10890080. ^48^ Data are available under the terms of the Creative Commons Zero “No rights reserved” data waiver (CC0 1.0 Public domain dedication).
