# Supplementary material for: Determinants of screening participation of disadvantaged populations in France: a qualitative study
Source: Open Res Eur. 2026 Oct 17;4:83. Originally published 2024 Apr 24. [Version 4] doi: 10.12688/openreseurope.17317.4 (PMC13555013; doi:10.12688/openreseurope.17317.4)
Supplement: Peer review history

# Reviewer report: Reviewer response for version 3

**DOI:** 10.21956/openreseurope.25034.r75801

**Published:** 2026-07-14

**Authors:** Angela Hassiotis

**Affiliations:** 1 Division of Psychiatry, University College London, London, England, UK

This study is one of the outputs from an EU Horizon 2020 and describes research that took place some years ago now. It is a qualitative project that engaged 30 participants from disadvantaged backgrounds in interviews about their participation in national screening programs about a variety of disorders in France. However, some of the findings and challenges are likely to have wider resonance.

 The authors have already responded to two rounds of revisions and in my view, they have already covered the points raised by the other reviewers adequately.

 My questions relate to the sample as follows:

 1. Where in France were these participants drawn from?

 2. What methods were used in order to identify and recruit them?

 3. The abstract, results section, it states that “People with disability reported to be more influenced by factors related to the healthcare domain” however, no specifics are given as to what those factors were in contrast to the individual and social domains. This would also need to be revised to add some indicative factors.

 Other than that, I do not have further comments.

If applicable, is the statistical analysis and its interpretation appropriate?

Not applicable

Is the study design appropriate and is the work technically sound?

Yes

Is the work clearly and accurately presented and does it engage with the current literature?

Yes

Are the conclusions drawn adequately supported by the results?

Yes

Are sufficient details of methods and analysis provided to allow replication by others?

Partly

Are all the source data and materials underlying the results available?

Yes

Reviewer Expertise:

Applied health research using mixed methods

I confirm that I have read this submission and believe that I have an appropriate level of expertise to confirm that it is of an acceptable scientific standard, however I have significant reservations, as outlined above.

# Response

**Published:** 2026-10-07

**Authors:** Alice Le Bonniec

**Affiliations:** Universite Claude Bernard Lyon 1, Villeurbanne, Auvergne-Rhône-Alpes, France

The authors are very grateful to the reviewers for their feedback and suggestions to improve the manuscript. Details of the changes made to the manuscript are listed below:

 This study is one of the outputs from an EU Horizon 2020 and describes research that took place some years ago now. It is a qualitative project that engaged 30 participants from disadvantaged backgrounds in interviews about their participation in national screening programs about a variety of disorders in France. However, some of the findings and challenges are likely to have wider resonance. The authors have already responded to two rounds of revisions and in my view, they have already covered the points raised by the other reviewers adequately. My questions relate to the sample as follows:

1. Where in France were these participants drawn from?

**Response: **The study was conducted in the Rhone district in France. Participants from deprived backgrounds or with disability were recruited via two public and four associative organizations providing social care services in this district.

 2. What methods were used in order to identify and recruit them?

**Response: **More information has been added: “A convenience sampling type was used. The interviewers were given access to the facilities used by the partner organizations and could approach potential participants during regular activities (e.g. food distribution). Sometimes, specific participants were identified by a healthcare professional or a social worker, and they were contacted directly by the research team if they gave their approval beforehand.”

1. The abstract, results section, it states that “People with disability reported to be more influenced by factors related to the healthcare domain” however, no specifics are given as to what those factors were in contrast to the individual and social domains. This would also need to be revised to add some indicative factors.

**Response: **We added the following indicative factors: “perceived risk; recommendation from a healthcare professional or an educator”.

 Other than that, I do not have further comments.

# Reviewer report: Reviewer response for version 3

**DOI:** 10.21956/openreseurope.25034.r75212

**Published:** 2026-06-10

**Authors:** Hollyanna Wilson

**Affiliations:** 1 University of Hertfordshire, Hatfield, UK

Overall, this is a well-written manuscript that has the potential to benefit a wide range of healthcare professionals and services through its indexing. The adoption of a horizontal, person-centred approach to this topic represents a novel and welcome contribution to the literature. However, it should be acknowledged that the framework underpinning the study was applied exclusively to the exploration of health screening practices among men. This limitation warrants explicit discussion, including consideration of the relative paucity of research in this area relating to women's health and the implications this may have for the transferability of findings.

 There are several areas where minor grammatical revisions are required, and some key terms would benefit from clearer definition. Structurally, however, the manuscript is coherent and well organised.

 The principal area for further development relates to the integration of relevant theoretical frameworks and governance perspectives throughout the manuscript. Greater engagement with concepts such as behaviour change theory, health literacy, trauma-informed practice, and psychological safety would strengthen both the introduction and discussion sections. Similarly, consideration of relevant policy and governance frameworks, such as the United Nations Convention on the Rights of Persons with Disabilities (UNCRPD), would enhance the broader contextualisation of the findings.

 The discussion would also benefit from further elaboration in several areas. A number of important points are raised but could be developed through deeper social, academic, and clinical commentary. Expanding these arguments would help to strengthen the manuscript's contribution to the field, maximise reader engagement, and increase its potential impact across research, policy, and practice contexts.

 Specific points for revisions:

 INTRODUCTION:

  - Define/give examples to contextualise the terms 'underserved' (give definition/examples), 'socio-economic inequalities' (give examples) and 'disability' (?mental/intellectual/physical).

 METHODS

  - More information on recruitment needed, how was this done and please specify the sampling type e.g. convenience/purposive/self-selection

 RESULTS

  - Figure 1 is excellent - very accessible.

 - Fix grammatical errors e.g. 'at the opposite'

 DISCUSSION

 - do not refer to individuals 'staying in good shape' - substitute for something like 'stay healthy/maintain good health'

  - Comment on demographic skewing of participants in the context of generalisability issues

 - Comment on the intersectionality between deprivation and disability - the paper presents them as mutually exclusive, comment on the implications for individuals with multiple co-occurring demographic risk factors

  -  communication and decision-making tools should be relevant and accessible - please use this term.

If applicable, is the statistical analysis and its interpretation appropriate?

Not applicable

Is the study design appropriate and is the work technically sound?

Yes

Is the work clearly and accurately presented and does it engage with the current literature?

Yes

Are the conclusions drawn adequately supported by the results?

Yes

Are sufficient details of methods and analysis provided to allow replication by others?

Yes

Are all the source data and materials underlying the results available?

Yes

Reviewer Expertise:

Communication, intellectual disability, accessible information.

I confirm that I have read this submission and believe that I have an appropriate level of expertise to confirm that it is of an acceptable scientific standard, however I have significant reservations, as outlined above.

# Response

**Published:** 2026-10-07

**Authors:** Alice Le Bonniec

**Affiliations:** Universite Claude Bernard Lyon 1, Villeurbanne, Auvergne-Rhône-Alpes, France

The authors are very grateful to the two reviewers for their feedback and suggestions to improve the manuscript. Details of the changes made to the manuscript are listed below:

 Overall, this is a well-written manuscript that has the potential to benefit a wide range of healthcare professionals and services through its indexing. The adoption of a horizontal, person-centred approach to this topic represents a novel and welcome contribution to the literature. However, it should be acknowledged that the framework underpinning the study was applied exclusively to the exploration of health screening practices among men. This limitation warrants explicit discussion, including consideration of the relative paucity of research in this area relating to women's health and the implications this may have for the transferability of findings.

**Response**: This limitation has been added in the strengths and limitations section: “the framework underpinning the study was previously applied mainly to the exploration of health screening practices among men. This may limit the transferability of the study findings”. However, our previous umbrella review exploring barriers and facilitators to participation in health screening revealed that the screening programmes that were the most studied in the literature were the ones for cervical and breast cancer (exclusively targeting women).

 There are several areas where minor grammatical revisions are required, and some key terms would benefit from clearer definition. Structurally, however, the manuscript is coherent and well organised.

**Response:** A full revision of the manuscript has been conducted.

 The principal area for further development relates to the integration of relevant theoretical frameworks and governance perspectives throughout the manuscript. Greater engagement with concepts such as behaviour change theory, health literacy, trauma-informed practice, and psychological safety would strengthen both the introduction and discussion sections. Similarly, consideration of relevant policy and governance frameworks, such as the United Nations Convention on the Rights of Persons with Disabilities (UNCRPD), would enhance the broader contextualisation of the findings.

**Response**: These suggestions have been added throughout the manuscript. We referred to the UNCRPD and introduced the concepts of behaviour change theory and health literacy in the introduction. We discussed trauma-informed practice and psychological safety in the “implications and perspectives” section.

 The discussion would also benefit from further elaboration in several areas. A number of important points are raised but could be developed through deeper social, academic, and clinical commentary. Expanding these arguments would help to strengthen the manuscript's contribution to the field, maximise reader engagement, and increase its potential impact across research, policy, and practice contexts.

**Response:** We elaborated further in the introduction and discussion using the concepts suggested by the reviewer.

 Specific points for revisions:

 INTRODUCTION:  - Define/give examples to contextualise the terms 'underserved' (give definition/examples), 'socio-economic inequalities' (give examples) and 'disability' (mental/intellectual/physical).

**Response: **We clarified what was included in the term “disadvantaged populations” that is used throughout the manuscript. In the interest of accuracy, we kept the terms that were used by the authors of the studies when we referred to them. In Barré et al (2016), the term “deprived” was used, implying various factors such as the density of gynaecologists and deprivation of the municipality of residence, according to the European Deprivation Index. Lang et al (2016) and Buron et al (2017) estimated the level of “socioeconomic deprivation” using respectively the Indices of Multiple Deprivation (IMD) scores and a calculation of deprivation quintiles. Jacomelli et al (2016) used the Index of Multiple Deprivation to compare screening rates between the most versus the least “deprived areas”. Broberg et al (2018) used the broader terms “socioeconomic and demographic variables” that consider cohabiting status, disposable family income, employment status, unemployment benefits, social benefits and education level.  We also specified that disability could be physical, psychic or intellectual.

 METHODS  - More information on recruitment needed, how was this done and please specify the sampling type e.g. convenience/purposive/self-selection

**Response: **More information has been added: “A convenience sampling type was used. The interviewers were given access to the facilities used by the partner organizations and could approach potential participants during activities (e.g. food distribution). Sometimes, specific participants were identified by a healthcare professional or a social worker, and they were contacted directly by the research team if they gave their approval beforehand.”

 RESULTS  - Figure 1 is excellent - very accessible. - Fix grammatical errors e.g. 'at the opposite'

**Response: **This error has been fixed, as well as other grammatical errors.

 DISCUSSION - do not refer to individuals 'staying in good shape' - substitute for something like 'stay healthy/maintain good health'

**Response: **This has been corrected.

  - Comment on demographic skewing of participants in the context of generalisability issues **Response: **We added this point to the limitations of the study.

 - Comment on the intersectionality between deprivation and disability

 - the paper presents them as mutually exclusive, comment on the implications for individuals with multiple co-occurring demographic risk factors

**Response: **We fully agree with this suggestion and we have commented on that point in the discussion.

  -  communication and decision-making tools should be relevant and accessible - please use this term.

**Response: **The term “accessible” has been added in the conclusion.

# Reviewer report: Reviewer response for version 2

**DOI:** 10.21956/openreseurope.23644.r64921

**Published:** 2025-12-26

**Authors:** Emma Parry

**Affiliations:** 1 Keele University, Keele, England, UK

The authors have largely addressed my comments. I would advise updating the model in Figure 1. I think this needs to be formatted better.

If applicable, is the statistical analysis and its interpretation appropriate?

Partly

Is the study design appropriate and is the work technically sound?

Yes

Is the work clearly and accurately presented and does it engage with the current literature?

Partly

Are the conclusions drawn adequately supported by the results?

Partly

Are sufficient details of methods and analysis provided to allow replication by others?

Partly

Are all the source data and materials underlying the results available?

Partly

Reviewer Expertise:

Primary care research, health inequalities, mixed methods

I confirm that I have read this submission and believe that I have an appropriate level of expertise to confirm that it is of an acceptable scientific standard.

# Reviewer report: Reviewer response for version 1

**DOI:** 10.21956/openreseurope.18717.r57431

**Published:** 2025-09-02

**Authors:** Emma Parry

**Affiliations:** 1 Keele University, Keele, England, UK

The authors tackle an important topic, which is exploring how people from deprived backgrounds and who have a disability approach screening and facilitators and barriers to screening. As people from these groups are less likely to participate in screening programmes it is crucial to explore reasons behind this. The article is well written.

 My comments/feedback are below:

**Methods**

- Did you use purposive sampling? Did the authors sample people who had and had not participated in screening
- The interviews were conducted in 2019, why have the results only been published now?
- Was  PPI group involved? Was the PPI group involved in interpretation of results and triangulation of data?

**Results**

- The authors describe how many participants had been involved in screening. What type of screening had they participated in?

I am not sure I agree with the interpretation with regards to the subheadings given some of  the text presented. I would advise the authors to either reconsider some of the subheadings or use different quotes to support them. For some of the points it would have been helpful for the authors to expand more on some of the quotes and to explore participants responses in further depth. A few examples are below:

- For the person who described not having the time to attend screening did they explain why this was? I am wondering given their socioeconomic position could they have been working 2 jobs, have caring responsibilities etc. I think this would have been important to explore further and present here in the results.
- I’m not sure I agree with labelling the of the “links with relatives and friends” subheading. For the person who described people around her who had become ill, surely she is describing here fear as a reason for screening?
- For the subheading of “Fatigue if too many medical procedures…” are the quotes also implying lack of knowledge of the positives for screening e.g. preventing disease before it becomes burdensome?
- I don’t think the subheading “Being followed for a disease/medical procedure” is correct. Do the authors mean followed-up? Also, the language used by the 42 year old women is interesting, she uses the word “forced” which is powerful. The authors might want to consider updating this interpretation of this subheading.
- For the subheading “difficulties linked with disability” I’m not sure the participants are describing this. One participant is talking about how her cervix is narrow so I’m not sure how this is linked to disability.

I would caution the authors against making inferences based on frequency counts when analysing qualitive data, for example, comparing number of barriers between participants with disability and deprived backgrounds.

 It would have been nice to have seen the results presented as a model at the end of the results section.

 Further information could have been given on the types of disabilities participants experienced to provide context to their responses.

**Discussion**

 The authors describe fear as a barrier to screening amongst deprived and disabled populations. It would be helpful to readers if the authors could expand on why they think fear is a barrier e.g. what are people fearful of?

 One limitation of the study, not discussed here is the study was limited to French speaking participants only. Also ethnicity of the participants is not reported. Surely this will have influenced findings?

 I found the implications section generic and did not feel this really took into account the findings from the study, nor did it go beyond practical suggestions that are likely to already be in place.

If applicable, is the statistical analysis and its interpretation appropriate?

Partly

Is the study design appropriate and is the work technically sound?

Yes

Is the work clearly and accurately presented and does it engage with the current literature?

Partly

Are the conclusions drawn adequately supported by the results?

Partly

Are sufficient details of methods and analysis provided to allow replication by others?

Partly

Are all the source data and materials underlying the results available?

Partly

Reviewer Expertise:

Primary care research, health inequalities, mixed methods

I confirm that I have read this submission and believe that I have an appropriate level of expertise to confirm that it is of an acceptable scientific standard, however I have significant reservations, as outlined above.

# Response

**Published:** 2025-11-05

**Authors:** Alice Le Bonniec

**Affiliations:** Universite Claude Bernard Lyon 1, Villeurbanne, Auvergne-Rhône-Alpes, France

The authors are very grateful to the two reviewers for their feedback and suggestions to improve the manuscript. Details of the changes made to the manuscript are listed below:

**Reviewer 2** The authors tackle an important topic, which is exploring how people from deprived backgrounds and who have a disability approach screening and facilitators and barriers to screening. As people from these groups are less likely to participate in screening programmes it is crucial to explore reasons behind this. The article is well written.  

 My comments/feedback are below:  

 Methods:

 Did you use purposive sampling? Did the authors sample people who had and had not participated in screening

**Response: **The only eligibility criteria was related to the disadvantaged background of participants (experiencing deprivation or having a disability). We did not sample people who had and had not participated in screening. This is justified by the difficulty of findings volunteers in these hard-to-reach populations. Actually, 28 out of 30 study participants had already participated in at least one screening. However, it is likely that only a few or none of them had participated to all screening recommended to them (now added in the limitations section).

 The interviews were conducted in 2019, why have the results only been published now?

**Response: **The results were already disseminated to a French audience, before making them available to a wider audience. As there is no staff working on this project anymore, the publication process relies on the authors availability which can be limited due to other work commitment, explaining that the manuscript was submitted in April 2024.  

 Was PPI group involved? Was the PPI group involved in interpretation of results and triangulation of data?

**Response: **No PPI group was involved in this study. This has been added to the limitations.

 Results:

 The authors describe how many participants had been involved in screening. What type of screening had they participated in? The participants took part in various screening procedures – the main screening procedures available in France were considered (please see appendix II for more details). The most frequent screening procedures reported by participants were cancer screening tests, diabetes tests and unspecified blood tests. I am not sure I agree with the interpretation with regards to the subheadings given some of the text presented. I would advise the authors to either reconsider some of the subheadings or use different quotes to support them. For some of the points it would have been helpful for the authors to expand more on some of the quotes and to explore participants responses in further depth. A few examples are below: For the person who described not having the time to attend screening did they explain why this was? I am wondering given their socioeconomic position could they have been working 2 jobs, have caring responsibilities etc. I think this would have been important to explore further and present here in the results.

**Response: **Regarding this specific interview, the participant expressed a lack of time due to her son being sick and needing frequent hospital appointments. We added some examples into brackets after the subheadings in Table 2 to illustrate the content of the interviews mentioning the lack of time as a barrier.

 I’m not sure I agree with labelling the of the “links with relatives and friends” subheading. For the person who described people around her who had become ill, surely, she is describing here fear as a reason for screening?

**Response: **We replaced this subheading by “social influences” as it reflects more accurately the quotes. Regarding the person who described people around her who had become ill, we cannot infer that she expresses fear but this is, at least, an expression of concern.

 For the subheading of “Fatigue if too many medical procedures…” are the quotes also implying lack of knowledge of the positives for screening e.g. preventing disease before it becomes burdensome?

**Response: **It is possible but we cannot infer the lack of knowledge. Participants might be aware of the benefits of the screening procedures but still feel it is cumbersome to do them all.

 I don’t think the subheading “Being followed for a disease/medical procedure” is correct. Do the authors mean followed-up? Also, the language used by the 42 year old women is interesting, she uses the word “forced” which is powerful. The authors might want to consider updating this interpretation of this subheading.

**Response: **Thank you for noting that mistake. We replaced “follow” by “follow-up”. We changed the subheading (now “Screening seen as inevitable when followed up for a disease/medical procedure”) and added a sentence in the discussion about the “forced” screening procedures.

 For the subheading “difficulties linked with disability” I’m not sure the participants are describing this. One participant is talking about how her cervix is narrow so I’m not sure how this is linked to disability.

**Response: **We replaced that subheading by “Screening procedure difficult to access/perform”.

 I would caution the authors against making inferences based on frequency counts when analysing qualitive data, for example, comparing number of barriers between participants with disability and deprived backgrounds. It would have been nice to have seen the results presented as a model at the end of the results section.

**Response: **We deleted the figure 1 (comparing number of barriers between the two populations) and replaced it by a model presenting the results.

 Further information could have been given on the types of disabilities participants experienced to provide context to their responses.

**Response: **As stated in the data collection section, participants were included if they were living with a physical or mental/intellectual disability.  More details on the disability of each participant were not collected in order to respect their privacy.  

 Discussion:

 The authors describe fear as a barrier to screening amongst deprived and disabled populations. It would be helpful to readers if the authors could expand on why they think fear is a barrier e.g. what are people fearful of?

**Response: **We expended on that topic in the discussion: “A distinction can be made between a fear of the results of the test (i.e. diagnosis, consequences of a disease) and a fear of the screening procedure (i.e. painful test) (Le Bonniec et al, 2022).”

 One limitation of the study not discussed here is the study was limited to French speaking participants only. Also ethnicity of the participants is not reported. Surely this will have influenced findings?

**Response: **We added these limitations.  

 I found the implications section generic and did not feel this really took into account the findings from the study, nor did it go beyond practical suggestions that are likely to already be in place.

**Response: **We added more suggestions in this section. The horizontal approach taken in this research aims to go beyond what is already implemented in France. The next steps are still in progress through the Lianeli project that aims to develop a digital application for people to access tailored information about screening (more information here: https://lianeli.fr/). A recent publication (Meunier et al, 2024) as part of this project showed the complexity of implementing a horizontal approach without standardised information integrated in current practice tools.

 Reference: Yves Meunier, P., Maiga, K., Maillet, D., Bochaton, T., De la Fournière, B., Delahaye, F., Dupont, C., Guillaud, O., Lamort-Bouché, M., Lina, B., Ray-Coquard, I., Péron, J. et Letrilliart, L. (2024). Recommandations de dépistage en France : une revue systématique. Santé Publique, . 36(5), 15-35. https://doi.org/10.3917/spub.245.0015.

# Reviewer report: Reviewer response for version 1

**DOI:** 10.21956/openreseurope.18717.r47682

**Published:** 2025-01-08

**Authors:** Johann Cailhol

**Affiliations:** 1 University Sorbonne Paris Nord, Seine Saint Denis University Hospital,, Bobigny, France

The authors conducted a qualitative study on barriers and facilitators of screening in general, in a sample of people living with disability and / or deprived. 

 While the article is well presented and easy to read, a number of improvements are needed before it could be officially index.

 My first main concern is the lack of linkage between the statements in the introduction, about the low literacy level in deprived population and its impact on screening uptake, and the methodology. If my understanding is correct, the literacy level has not been questioned in the interview, which is a shortcoming. In the same vein, authors refer to 2 papers on the low screening uptake among very specific migrant categories (homeless or deprived migrants), who cumulate barriers to screening but are not representative of other categories of deprived / disabled population in France. 

 Introduction / context sections could therefore be improved by including more general figures on the lowest SES quartile of French population accessing screening, compared to the highest quartile or average. 

 My second main concern lies in the methodology. Authors used the framework by Teo and al in order to conduct their interviews (and therefore the framework analysis). While i would have liked to read more in detail on Teo's framework, i can't see specific questions on financial or geographical barriers (related to transportation for instance), that could be experienced by the specific populations under scrutiny.

 The findings section could be improved by going deeper in the interpretations of interviews. For instance, the perception of "imposed screening" by the 2 women (disabled, 34yo and deprived, 42yo), merits greater attention. 

 The discussion should make the link with pre-existing horizontal screening campaigns such as the one organized by CPAM, especially targeting deprived population.

 What about including occupational medicine for short-term contracted workers, or public services such as France Travail, or the CAF, or MDPH, in order to systematically reach the most deprived?

If applicable, is the statistical analysis and its interpretation appropriate?

Partly

Is the study design appropriate and is the work technically sound?

Yes

Is the work clearly and accurately presented and does it engage with the current literature?

Partly

Are the conclusions drawn adequately supported by the results?

Partly

Are sufficient details of methods and analysis provided to allow replication by others?

No

Are all the source data and materials underlying the results available?

No

Reviewer Expertise:

Health systems and policies; migrant health; health litteracy

I confirm that I have read this submission and believe that I have an appropriate level of expertise to state that I do not consider it to be of an acceptable scientific standard, for reasons outlined above.

# Response

**Published:** 2025-11-05

**Authors:** Alice Le Bonniec

**Affiliations:** Universite Claude Bernard Lyon 1, Villeurbanne, Auvergne-Rhône-Alpes, France

The authors are very grateful to the two reviewers for their feedback and suggestions to improve the manuscript. Details of the changes made to the manuscript are listed below:  

 The authors conducted a qualitative study on barriers and facilitators of screening in general, in a sample of people living with disability and / or deprived.

 While the article is well presented and easy to read, a number of improvements are needed before it could be officially index. My first main concern is the lack of linkage between the statements in the introduction, about the low literacy level in deprived population and its impact on screening uptake, and the methodology. If my understanding is correct, the literacy level has not been questioned in the interview, which is a shortcoming. In the same vein, authors refer to 2 papers on the low screening uptake among very specific migrant categories (homeless or deprived migrants), who cumulate barriers to screening but are not representative of other categories of deprived / disabled population in France.

**Response:  **We deleted the sentence linking health literacy level and screening uptake. In addition, we replaced the citations related to migrants populations by another paper including wider deprived populations (Sassenou et al, 2025), and we now cite additional French studies including more representative populations (Barre et al 2017; Ouanhnon et al 2022 ). The fact that the level of health literacy was not assessed during the interviews was added as a limitation.

 Introduction / context sections could therefore be improved by including more general figures on the lowest SES quartile of French population accessing screening, compared to the highest quartile or average.

**Response:  **Figures on the participation in screening within different French areas are not always accessible to illustrate screening disparities but we added data on cervical and breast screening participation as an example (Barre et al, 2017; Ouanhnon et al, 2022).

 My second main concern lies in the methodology. Authors used the framework by Teo and al in order to conduct their interviews (and therefore the framework analysis). While i would have liked to read more in detail on Teo's framework, i can't see specific questions on financial or geographical barriers (related to transportation for instance), that could be experienced by the specific populations under scrutiny.

**Response: **We added some examples for each domain of Teo’s framework in the data analysis section.  The framework was used to analyse the results of the interviews. This framework includes indeed the possible lack of income or personal insurance and the lack of personal transport. The semi-structured interviews were based on open-ended questions – e.g. “Is there anything that prevent you from participating in screening?”. That way any potential barriers could be raised by participants. Financial aspects were not mentioned as a barrier by participants. This can be explained by the fact that screening is free of charge in France. Geographical factors did not appear as a barrier either – this is probably due to the fact that people included in the interviews were living in a big city with short distances to healthcare facilities and easy access to public transports.

 The findings section could be improved by going deeper in the interpretations of interviews. For instance, the perception of "imposed screening" by the 2 women (disabled, 34yo and deprived, 42yo), merits greater attention.

**Response: **We elaborated on this point in the discussion: “Indeed, two participants expressed a perception of imposed screening (blood tests) as part of another medical procedure. This specific context places screening as an inevitable step to access other care, whereas informed consent should be checked for each separate procedure.” The discussion should make the link with pre-existing horizontal screening campaigns such as the one organized by CPAM, especially targeting deprived population.

 What about including occupational medicine for short-term contracted workers, or public services such as France Travail, or the CAF, or MDPH, in order to systematically reach the most deprived?

**Response: **These elements have been added to the discussion.    
